# Effect of VUV Radiation on Surface Modification of Polystyrene Exposed to Atmospheric Pressure Plasma Jet

**DOI:** 10.3390/polym12051136

**Published:** 2020-05-15

**Authors:** Rok Zaplotnik, Alenka Vesel

**Affiliations:** Department of Surface Engineering, Jozef Stefan Institute, Jamova cesta 39, 1000 Ljubljana, Slovenia; rok.zaplotnik@ijs.si

**Keywords:** atmospheric-pressure plasma jet, VUV interaction, polymer polystyrene, evolution of wettability patterns, evolution of functional groups

## Abstract

Precise tailoring of surface properties by gaseous plasma treatments remains a key scientific challenge, especially when adequate surface wettability should be laterally distributed, and sharp interfaces between hydrophobic and hydrophilic areas are desirable. The evolution of surface wettability and functional groups on polystyrene (PS) upon treatment with argon plasma jet was monitored by water contact angles and X-ray photoelectron spectroscopy (XPS). An array of water droplets was deposited on PS samples treated either directly by the plasma jet or only VUV radiation arising from the plasma. Rather sharp interfaces between the activated and not-affected regions were observed in both cases. The functionalization with highly-oxidized carbon functional groups, as determined by high-resolution C1s XPS spectra, was by far more efficient using the VUV radiation only. In contrast, the optimal wettability was achieved using direct plasma treatment. The results were explained by different mechanisms involved in the interaction of radiation and reactive plasma species with the polymer surface.

## 1. Introduction

Polystyrene (PS) is among the most commonly used aromatic polymers. It is widely used in different industrial branches, and one of its important applications is in biology for multi-well plates. Because PS is a pure hydrocarbon material, it is often also used as a model polymer in studying surface functionalization. Functionalization is desirable in cases where the original wettability is not sufficient. A variety of functional groups causes improved wettability, but usually, authors employ oxygen-rich functional groups for increasing the surface energy. It is often important to assure for uniform functionalization of a polymer sample over a large surface area. In such cases, low-pressure oxygen plasma has been proved to cause sufficient wettability on large samples. An investigation of the interaction between low-pressure oxygen plasma and PS foils revealed some interesting results [1]. For example, it has been shown that treatment by oxygen glow plasma itself does not assure optimal functionalization. The flowing afterglow enabled almost double concentration of oxygen on the surface of PS as compared to the treatment with glowing plasma [1]. The difference between gaseous plasma and the afterglow is in the composition as well as radiation: Plasma itself is a source of VUV radiation arising from transitions of excited oxygen atoms to the ground state, and also a source of radiation in the red and near IR part of the spectrum, whereas only long-living radicals persist in the afterglow.

Polymers are often saturated with functional groups even after a short exposure to either oxygen plasma or its afterglow providing the flux of reactive species is large. The initial stages in the functionalization of PS using only oxygen atoms were investigated in the late afterglow chamber connected to an XPS instrument [2]. It was shown that the fluences of the O atoms as low as 10^22^ m^–2^ enable rich surface chemistry. Degradation of the aromatic ring was already observed at much lower fluences. The first functional group that appeared on the PS surface was the OH group. After prolonged treatment (i.e., larger O-atom fluences), other functional groups appeared on the surface, until the surface was fully saturated what occurred after receiving the O-atom fluence of approximately 10^23^ m^–2^.

Despite the fact that PS can be activated easily with desired functional groups in low-pressure plasma or afterglow, there is an increasing interest in the application of atmospheric pressure plasmas. One reason is just practical—it may be easier to perform experiments at ambient conditions. More scientific reasons include the requirement of laterally distributed functional groups. Such a lateral distribution is difficult to achieve in a low-pressure system because of extensive diffusion. The gradients of oxygen atoms in low-pressure systems are marginal as compared to atmospheric pressure systems [3] because of a lower probability for three-body collisions. The atmospheric pressure plasma jets (APPJs), therefore, represent rather well-collimated sources of reactive species useful for distributing the surface functional groups laterally as desired for specific applications. Typical cross-sections of plasma jets are of the order of mm^2^, but capillary discharges can also provide more focused jets [4].

Treatment of PS by APPJ has attracted attention from both industrial users and scientists. Oehrline et al. investigated etching of PS by an Ar + 1% H_2_O APPJ jet [5]. They found relatively high etch rates and weak oxidation of the surface. Etching rates decreased exponentially with increasing the distance between the APPJ nozzle and the polymer surface. This was in correlation with a change of the density of OH radicals with the distance; therefore, the authors concluded that OH radicals are crucial in PS etching. The authors compared the results obtained for Ar/H_2_O plasma with those for the Ar + 1% O_2_ plasma [6]. The etching rates for the Ar/H_2_O plasma, i.e., the number of carbon atoms removed per OH radical, were of the order of 10^–2^ [5]. In contrast, the etching probability of oxygen atoms in the Ar/O_2_ plasma was only 10^–4^ [6]. The authors also investigated the effect of the sample temperature on the etching rates by controlled heating or cooling the sample in the range between 0 and 90° [5]. They found that for the Ar/H_2_O plasma, the etching rates were higher at lower temperatures, whereas an opposite trend was observed for the Ar/O_2_ plasma. This observation was explained by the enhanced adsorption and different sticking coefficient of gaseous species such as OH and H_2_O at low temperatures. Another observation reported by the same authors is the influence of the composition of the surrounding atmosphere on the surface finish [7]. The etching rates in pure N_2_ surrounding-gas environment were much larger, whereas the surface was much less oxidized than in the case of the N_2_ + O_2_ environment.

The same group also investigated the modification of PS under remote conditions, where the plasma jet was not visible [7] and found negligible etching. The concentration of functional groups as probed by XPS strongly depended on the ambient and feed gas compositions. The concentration of oxygen in the feed gas had a significant effect on the surface finish. As expected, when oxygen was present in the feed gas, the surface was highly oxidized. Highly oxidized treatments caused the disappearance of aromatic carbon peak in the XPS C1s spectra. The authors also concluded that long-lived species such as singlet delta oxygen, high-energy photons, or NO_x_ were more important species for surface modification than O atoms or O_3_ molecules.

Another interesting investigation was performed by Bradley et al. [8]. Opposite to the conventional approach where the APPJ plasma jet is aligned vertically to the surface, the authors treated PS with the He plasma jet that was aligned parallel to the surface (side-on treatment). The axis of the plasma jet was 3 mm above the surface. They found a different degree of surface modification when the flow was laminar (that is close to the exit of the capillary discharge tube) or turbulent. Substantial decrease of a water contact angle (WCA) occurred only in the downstream region where the flow was turbulent, whereas laminar flow caused only minor surface changes. They concluded that the turbulent flow contains a significant amount of excited air species from the surrounding atmosphere, which are mixed with the He gas.

Fricke et al. [9] compared the APPJ treatment of PS with the low-pressure O_2_ microwave (MW) plasma treatment and found similar wettability when using the Ar/O_2_ feed gas for the APPJ treatment. The wettability of PS treated with the pure Ar APPJ plasma was slightly worse than for the Ar/O_2_ APPJ plasma. 

Dowling et al. [10] have investigated the surface modification of PS as a function of plasma cycle time, which was a measure of plasma intensity. The feed gas was a dry compressed air. After treatment, the water contact angle decreased from 93° to about 20°–30°. They also measured the temperature of the ceramic holder. At high plasma intensity, the temperature in the center that coincided with the axis of the plasma jet was over 80°. A radial gradient in the temperature was observed—15 mm from the center it dropped to 40°.

Oehrlein [11] also stressed the influence of UV or VUV radiation on the surface chemistry by comparing four different configurations of APPJ sources. The radiation arising from APPJ sustained in argon is extensive in the VUV range, where a broad continuum was observed in the range of wavelengths between approximately 120 and 130 nm. The source of such radiation was Ar_2_^*^ as confirmed by the systematic experiments by Golda et al. [12]. No author, however, performed systematic research on the roles played by reactive plasma species and radiation. In the present paper, we show the evolution of surface wettability, as well as functional groups on the PS surface versus treatment time by treatment either with an APPJ sustained in Ar or with VUV radiation arising from such plasma.

## 2. Materials and Methods

### 2.1. Plasma Treatment

**Figure 1:**
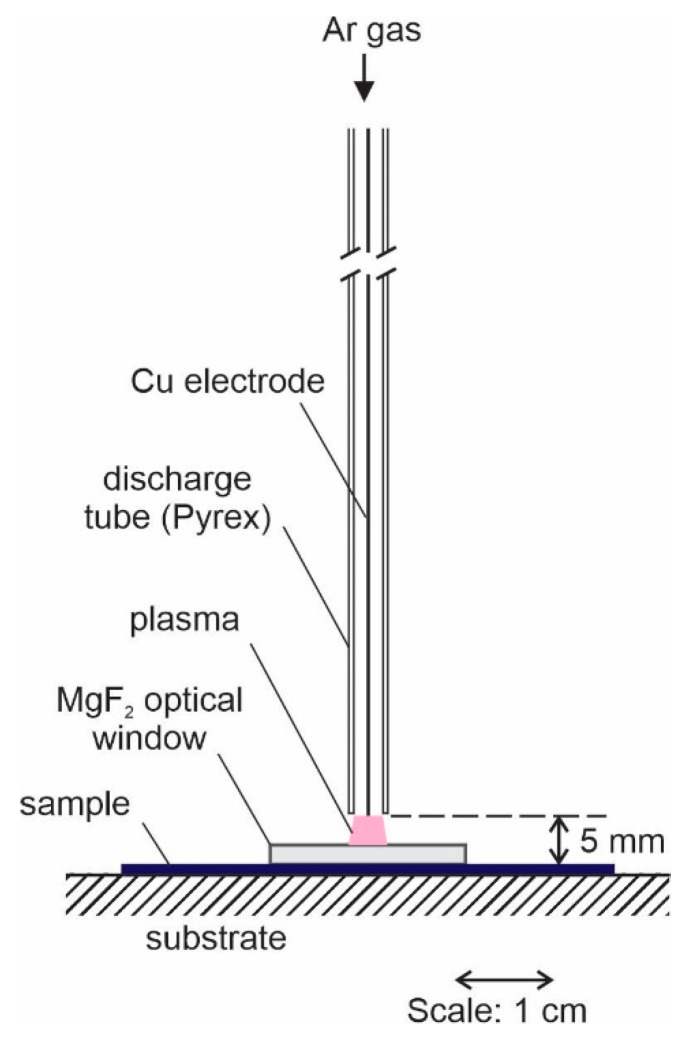
Schematic illustration of the experimental setup.

High purity polystyrene foils were purchased from Goodfellow Ltd (Huntingdon, England). No pretreatment was performed, but the foils were cut to square pieces of about 5 × 5 cm^2^ and positioned perpendicularly to a home-made APPJ device so that the center of the sample was at the axis of the APPJ device. The schematic of the experimental setup is shown in Figure 1. 

The system was kept at ambient conditions, i.e., air at atmospheric pressure, room temperature, and humidity of 60%. A single electrode was powered with a sinusoidal voltage source operating at a frequency of 25 kHz and a peak-to-peak voltage of 7 kV. Ar of purity 99.99% was introduced into the dielectric tube through a flow controller. The inner diameter of the dielectric tube was 3 mm. The electrode was a copper wire with a diameter of approximately 0.3 mm. The cross-section of the dielectric tube was therefore much larger than the cross-section of the Cu electrode. The Ar gas flow Φ_v_ was adjusted to 1 slm. The speed of gas at the exhaust of the dielectric tube was estimated from the cross-section of the tube and the gas flow as *v* = Φ_v_/*S*, where *S* is the cross-section area of the discharge tube. Considering numerical values, i.e., Φ_v_ = 1 L/min, *S* = 7 mm^2^, the velocity of the gas at the exit of the dielectric tube is approximately 2 m/s. The velocity is high enough to prevent significant diffusion of surrounding air inside the Ar jet along the axis. The PS foil was placed on the substrate holder 5 mm below the exit of the dielectric tube. The substrate was made from a dielectric (wood) and placed far from any metallic element. The sample was therefore at a floating potential. Some experiments were performed by direct exposure of the polymer foil to APPJ plasma, whereas in other cases, we placed the MgF_2_ optical window on the polymer surface, as shown in Figure 1. The MgF_2_ window (from Crystal GmbH, Berlin, Germany) was in the form of a circular disk with a thickness of 2 mm and a diameter of 2 cm. The MgF_2_ window is transparent for a broad range of UV radiation down to approximately 115 nm [13]. The MgF_2_ optical window served as material transparent for UV/VUV radiation but prevented direct contact between charged or neutral species, which may abound in gaseous plasma.

### 2.2. Water Contact Angle (WCA) Measurements

A mapping of the surface wettability of PS samples was performed with Drop Shape Analyser DSA-100 (Krüss GmbH, Hamburg, Germany). A static contact angle was measured using a sessile drop method. An array of 73 water drops with a volume of 1 µL was applied over the whole surface what enabled obtaining 2D images of the surface wettability. A distance between drops on the surface was 5 mm. A time elapsed between the first and the last measured drop was less than 13 min. More details about these measurements can be found in [14].

### 2.3. X-ray Photoelectron Spectroscopy (XPS) Measurements

Immediately after the APPJ treatment, the samples were transferred to the XPS chamber. XPS characterization of PS was performed using an XPS instrument (model TFA XPS from Physical Electronics, Münich, Germany). The samples were exposed to monochromatic Al Kα_1,2_ radiation at 1486.6 eV. The diameter of the measured area was 400 µm. Spectra were measured at an electron take-off angle of 45° in the center of the treated samples. Survey spectra were acquired at a pass-energy of 187 eV using an energy step of 0.4 eV, whereas high-resolution C1s spectra were measured at a pass-energy of 23.5 eV using an energy step of 0.1 eV. An additional electron gun was used for compensation of the surface charge. Spectra were calibrated by shifting the C–C peak to 284.8 eV. The measured spectra were analyzed using MultiPak v8.1c software (Ulvac-Phi Inc., Kanagawa, Japan, 2006) from Physical Electronics, which was supplied with the spectrometer. Linear background subtraction was used. The following peaks were identified in C1s spectra: C–C (284.8 eV), C–O (286.2 eV), C=O, O–C-O (287,4 eV), O–C=O (288.6 eV), as well as O–CO–O (289.7 eV) and aromatic shake-up peak π–π* (291.5 eV).

## 3. Results and Discussion

### 3.1. Evolution of Surface Wettability

**Figure 2:**
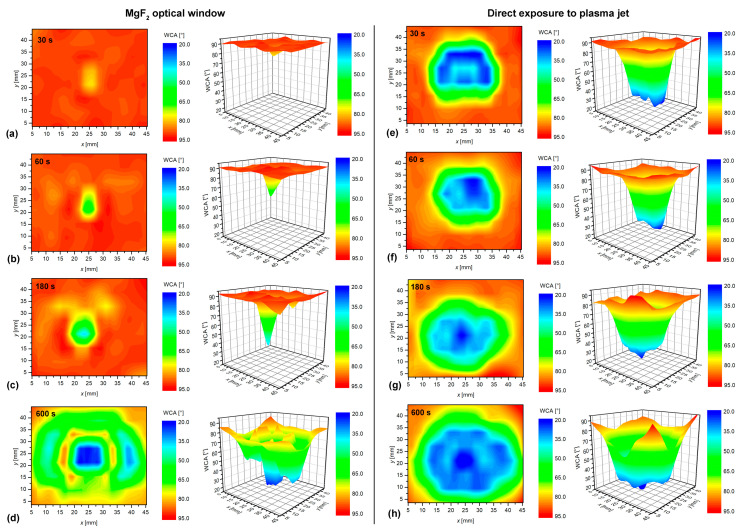
2D and 3D images of surface wettability of polystyrene (PS) samples treated for various treatment times 30, 60, 180, and 600 s: (**a**–**d**) images on the left are for the case when samples were covered with MgF_2_ window and thus exposed to VUV radiation only, whereas (**e**–**h**) images on the right are for the case of direct exposure to plasma (without any covering).

Polymer treatments were performed at a constant distance between the exit of the dielectric tube and the polymer surface of 5 mm and for various treatment times. Just after the treatment, the samples were probed for wettability using our multi-droplet device. The results are shown graphically in Figure 2.

The left column in Figure 2 is a 2D/3D plot of water contact angles for the case when PS samples were covered with the MgF_2_ optical window and thus exposed to UV-VUV radiation only. The right column in Figure 2 represents results without the MgF_2_ window when the samples were exposed directly to the plasma jet. Treatment times in all rows in Figure 2 were the same for covered and uncovered samples. At a glance, one can observe a saturation of the surface wettability for the case of direct plasma treatment, and a graduate increase of the surface wettability in the case of treatment by VUV radiation only. The water contact angle of the untreated PS was approximately 90°. The treatment by UV-VUV radiation for 30 s (Figure 2a) revealed a rather small spot of activated PS right in the center. As shown in Figure 1, the sample was positioned in such a way that it was centered on the axis of the discharge tube. The yellow spot on the surface treated for 30 s appeared at the position of the plasma jet. As mentioned earlier, the inner diameter of the dielectric tube is approximately 3 mm, and so is the diameter of the activated spot on the PS treated by the radiation only for 30 s. This observation indicates that the radiation suitable for surface activation is focused in the same way as plasma itself. The radiation, of course, occurs over the entire length of the plasma jet, and the excited atoms or molecules radiate in all directions. The fact that the activated spot is rather well-focused indicates that the radiation that should pass the effluent region away from the axis of the gas jet does not contribute to surface activation. This is a consequence of a rather short absorption length, which is of the order of 100 μm [11]. 

A double treatment time (60 s) shows improved wettability, as indicated in Figure 2b. The diameter of the activated spot remains practically the same, but the water contact angles at the center of the sample are much smaller. The interface between the region of no activation and the area with low water contact angles is very sharp. The reason is probably the poor transparency of the ambient atmosphere for radiation arising from gaseous plasma.

The prolonged treatment by radiation, i.e., after 180 s, causes a further increase of the surface wettability in the center of the activated spot, as revealed in Figure 2c. Simultaneously, the spot size increases, indicating that the VUV radiation is not perfectly focused. Such a long treatment time is, therefore, sufficient for increasing the size of the activated spot over the diameter of the dielectric tube. Weakly activated areas are also observed outside the spot. The appearance of such rather unexpected observation is explained by considering the result of PS treated for 600 s, which is shown in Figure 2d. As already mentioned, the MgF_2_ window was smaller than the size of the sample; therefore, only the central part was covered. In Figure 2d, we observe a central spot of a well-activated PS and a halo stretching far away from the axis. In-between, there is a ring-like area of poor wettability. The diameter of the ring coincides with the diameter of the MgF_2_ window. The spot of the well-activated surface, which was fully covered with the MgF_2_ window, is obviously because of a large fluence of VUV radiation. However, the halo formed on the uncovered PS area is probably because of the interaction of reactive chemical species present away from the axis of the experimental setup that may rich the uncovered PS surface. The halo is therefore explained by weak activation of air molecules away from the main Ar jet.

The right-hand column in Figure 2e–h shows a less pronounced evolution of the surface wettability with treatment time when no optical covering was used. Still, the area of the low water contact angle after 600 s of treatment (Figure 2h) is approximately double as compared to 30 s (Figure 2e). The results show that the minimum achievable contact angle of approximately 13° is obtained on the spot much larger than the jet diameter already after 30 s of plasma treatment. The prolonged treatment causes enlargement of the spot size, but the interface between non-treated and well-activated surface remains fairly unchanged. Such observation is typical for the evolution of any surface property where saturation occurs.

### 3.2. Evolution of Chemical Modifications

The samples whose wettability was elaborated in Figure 2 were also characterized by XPS. Because of the time-consuming XPS measurements, the XPS spectra were acquired only in the center of the polymer sample, which was aligned with the axis of the APPJ jet. The XPS surface composition versus treatment time is presented in Table 1 and Table 2. 

Table 1 represents the surface composition versus treatment time for the case of samples covered with the MgF_2_ window. Even the untreated PS contains about 2 at. % of oxygen, which is probably because of the weak oxidation of the polymer foil before plasma treatment. The treatment for 30 s shows enrichment of PS surface with oxygen as well as some nitrogen. The concentration of oxygen increases with increasing treatment time, as indicated in Table 1. The increase is rather monotonous. The final concentration of 30 at. % of oxygen is comparable to the best results observed by low-pressure oxygen plasma treatment [2,15]. The increase of oxygen concentration versus treatment time is in agreement with the surface wettability shown in Figure 2a–d, where we can also observe a gradual increase with treatment time.

**Figure 3:**
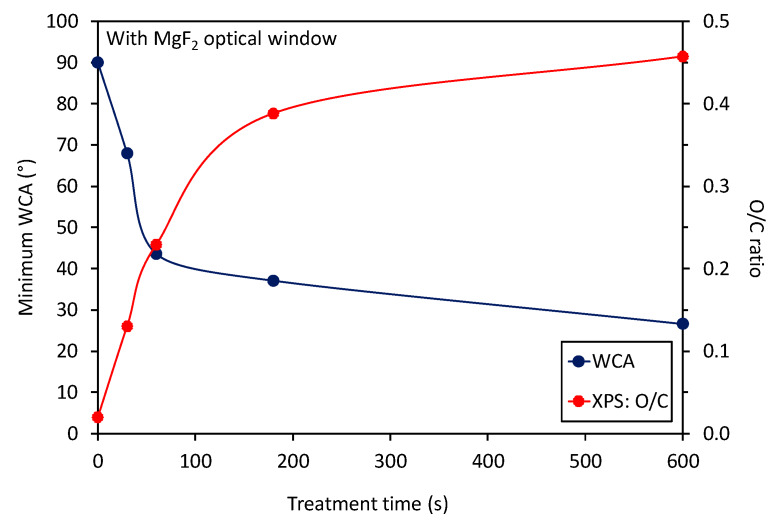
Variation of the minimum water contact angle and O/C ratio versus treatment time for the case when samples were covered with the optical window.

**Figure 4:**
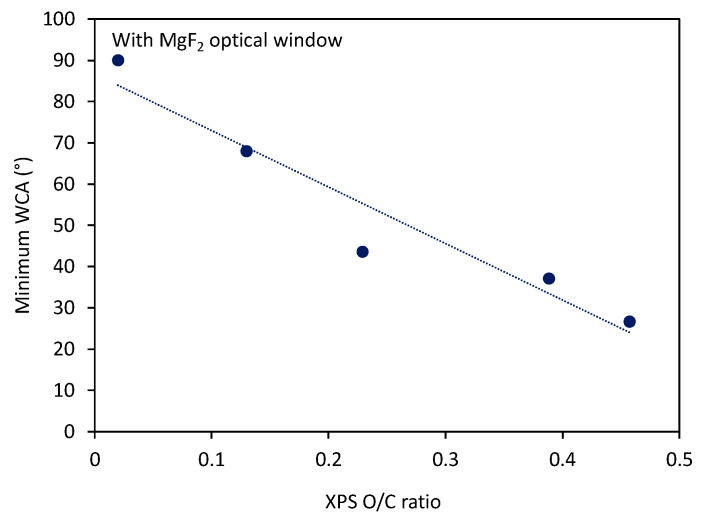
Correlation between the minimum water contact angle and O/C ratio for the case when samples were covered with the optical window.

Interesting is the evolution of the O/C ratio of samples treated with radiation only versus the treatment time, which is shown in Figure 3. One can observe a highly monotonous approaching of saturation, which occurs at the O/C ratio close to 0.45. In the same figure, there is also the curve for the minimum WCA measured in the center of the PS samples. The trends of WCA and surface composition are opposite as expected and reported already by other authors [16,17,18]. Additionally, in Figure 4 is shown a correlation between the minimum WCA versus the XPS O/C ratio. One can observe a monotonous decrease. In Figure 4, we used a linear fitting of the measured points for eye guidance only. 

**Figure 5:**
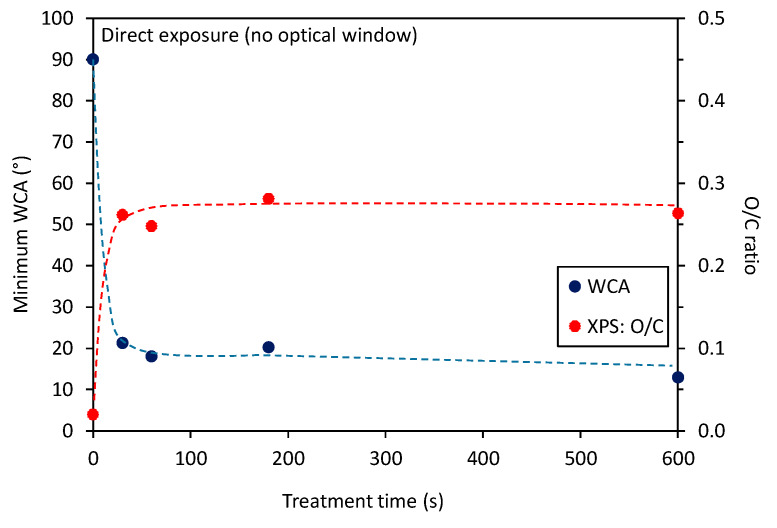
Variation of the minimum water contact angle and O/C ratio versus treatment time for the case of direct exposure of samples to plasma jet (no optical covering).

Table 2 represents the concentration of different elements in the center of the samples treated directly by the plasma jet. Opposite to results presented in Table 1, both oxygen and nitrogen concentrations in Table 2 remained practically unchanged with treatment time. This observation is sound with the right column of Figure 2e–h, which shows the saturation of surface wettability in sample’ centers already at the shortest treatment time. What is more interesting is the fact that the observed O/C ratio in Table 2 is significantly lower than in Table 1. The graphical presentation of the results of surface functionalization and wettability versus the treatment time is shown in Figure 5. One can observe almost perfect saturation of both surface properties. The rather poor oxidation of the PS sample upon direct exposure to APPJ as compared to treatment by VUV radiation is worth discussing. Such an observation was already reported by Oehrline [7] where better functionalization was observed for samples treated by remote plasma jet, i.e., outside the glowing plasma. The authors [7] concluded that long-lived species, such as singlet oxygen molecules, may be more important for surface activation than short-lived species such as O atoms. Furthermore, they proposed an important effect of high-energy photons. The same group also reported etching upon treatment of PS with plasma rich in reactive species [6]. The latter effect can explain a rather poor surface functionalization when using direct plasma treatment, as observed in our case. As is already known for low-pressure plasma, the plasma species cause both chemical and physical interaction with the polymer surface [15]. Excessive energy stimulates more extensive chemical reactions, which probably results in the formation of oxygen-rich molecular fragments, which may desorb from the surface. This effect can be avoided efficiently in the case of low-pressure treatment by using late afterglows instead of gaseous plasma. In the case of a direct treatment by plasma jet, the mechanisms of interaction between O atoms and polymer surfaces are difficult to evaluate because of the huge gradients of reactive species. 

The treatment by VUV radiation from the APPJ is more efficient than by direct plasma treatment as long as the concentration of oxygen, as determined by XPS, is the merit. The VUV is known to break bonds in organic materials, and dangling bonds are attacked by gaseous molecules [19]. Dangling bonds are very reactive, so even exposure to molecular oxygen or nitrogen causes functionalization. Interestingly enough, the water contact angle remains rather large even after prolonged treatment. The WCA for samples treated directly by plasma jet stabilizes at about 20° (Figure 5), whereas for VUV treatment it keeps decreasing slowly, but remains over 25° even after 10 min (Figure 3). Here it is worth mentioning that the surface wettability reflects both the surface functionalization and roughness, whereas XPS reveals only somehow averaged concentration of an element over the probing depth. While plasma species do not penetrate deep into the polymer, the VUV radiation has a penetration depth typically much larger than the probing depth of XPS [20]. One possible explanation of the paradox could be the modification of a thicker surface film by the VUV. Although VUV has been proved to cause also etching [19], the etching rate is much lower than for the case of etching with plasma radicals. Such a rather poor etching enables the preservation of highly saturated functional groups on the polymer surface and thus the ability for better functionalization of the subsurface layer. Another possible explanation of the paradox is different surface roughness. It was already shown that etching causes higher surface roughness [6], and an increase in roughness has an effect on the surface wettability [21].

**Figure 6:**
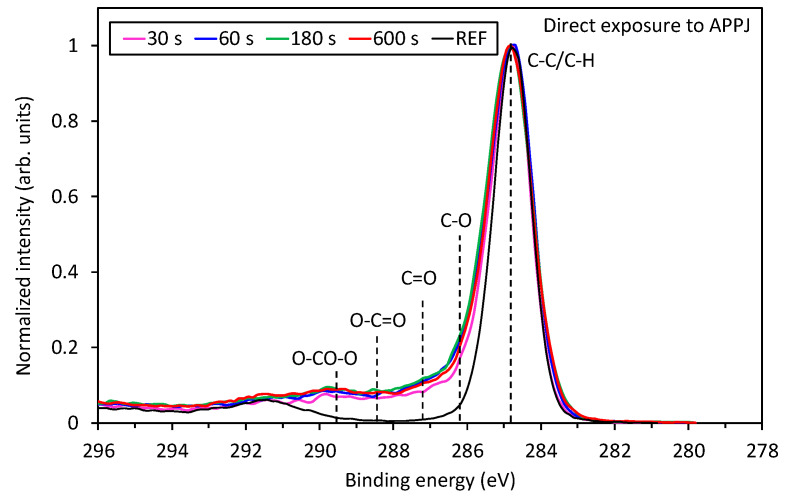
Comparison of high-resolution XPS C1s spectra of samples exposed directly to Ar plasma jet for various treatment times.

**Figure 7:**
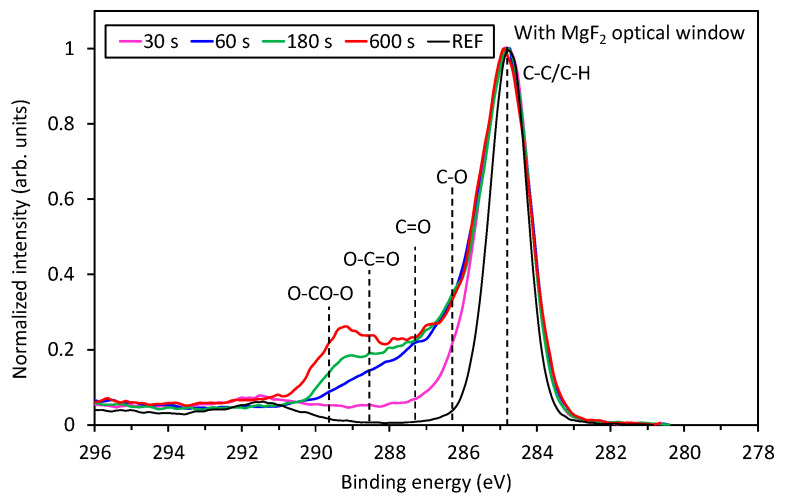
Comparison of high-resolution XPS C1s spectra of samples covered with MgF_2_ optical window and thus treated with VUV radiation for various treatment times.

Different evolutions of XPS oxygen concentration shown in Table 1 and Table 2 are reflected in a different degree of surface functionalization of samples treated by VUV or by direct plasma treatment. The evolution of surface functional groups is revealed from the high-resolution C1s spectra of samples shown in Figure 6 and Figure 7. Figure 6 represents the high-resolution XPS spectra of samples treated by direct exposure to the plasma jet. The untreated sample shows only one peak at about 285 eV attributed to C–C/C–H. Another peak at 291.5 eV corresponds to the shake-up peak reflecting the aromatic character of this polymer. The high-resolution spectra were acquired for all treatment times. One can observe almost perfect overlapping of the curves. The result is expected, taking into account the saturation of the polymer surface as revealed from Table 2 and Figure 5, as well as Figure 2e–h. Interestingly enough, no specific functional group predominates on the surface of the samples treated directly by APPJ.

**Figure 8:**
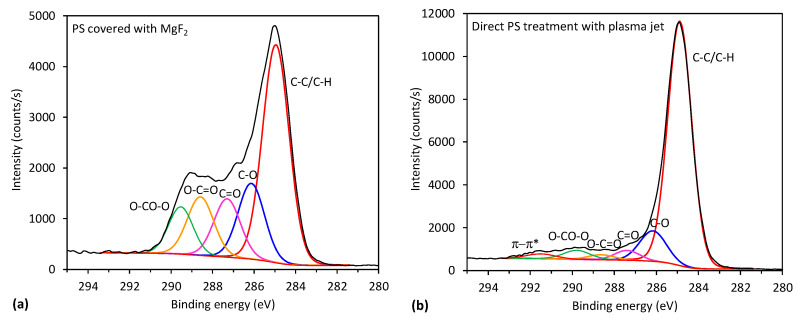
XPS C1s spectrum with subcomponents for PS treated for 10 min by: (**a**) VUV radiation and (**b**) by direct plasma jet.

More interesting is the evolution of the surface functionalities for samples treated by VUV radiation only, which is shown in Figure 7. Treatment by 30 s definitely causes some changes, but they are rather similar to those observed in Figure 6. However, 1 min of treatment already reveals a substantial formation of highly-oxidized functional groups. Finally, after 10 min of VUV radiation, a well-distinguished peak occurs at the binding energy of ~289 eV, which corresponds to carboxyl and carbonate groups, as explained below. This significant difference between Figure 6 and Figure 7 regarding the evolution of functional groups on the PS sample treated by VUV radiation or treated directly by APPJ is further supported by Figure 8 and Table 3. In Figure 8 is shown a comparison of a fitting of C1s peaks for the longest treatment time (10 min) for both treatment conditions, where the differences are the most noticeable. As already mentioned above, high concentrations of highly oxidized carbon functional groups are formed for the case of VUV treatment. More detailed evolution of specific functional groups for the samples treated by VUV radiation is shown in Table 3. For comparison, also direct plasma treatment is shown, but only for 10 min of treatment because the surface is already saturated at short treatment times (Figure 6). In Table 3, we can observe significant destruction of the aromatic ring in the case of VUV treatment, which is related to the formation of highly oxidized carbon groups. Such aromatic ring opening and formation of carbonate groups for PS have already been reported in the literature for low-pressure plasma treatments [2,15] and for the remote (afterglow) APPJ treatment [7], even though it is easier to oxidize and break the aliphatic chain than the aromatic one.

The effect of PS treatment by VUV radiation is thus more similar to the effect of low-pressure gaseous plasma (or afterglow) treatment than to APPJ [1,2,15]. The possible explanation of this observation is that the power density at atmospheric pressure plasma is by far larger than in low-pressure plasma useful for surface activation of polymers. According to [1,2,15], the best results in terms of surface activation of polymers by low-pressure plasma treatment are observed using weakly ionized gaseous plasma such as electrodeless RF discharge in the capacitive mode. In such plasma, the kinetic effects are entirely negligible as compared to chemical effects (as long as the samples are kept at a floating potential), because the dissociation fraction is orders of magnitude larger than ionization. Highly ionized low-pressure plasmas do not allow for optimal surface functionalization due to extensive etching. Taking into account these considerations, the rather poor functionalization of PS samples upon direct exposure to glowing APPJ (Figure 6) is a consequence of the simultaneous etching of highly polar oxygen compounds.

## 4. Conclusions

The influence of the gaseous plasma species and VUV radiation on the surface finish of the polystyrene as studied by water contact angles and XPS reveals the applicability of such a simple technique for selective surface activation of products made from this polymer. Rapid functionalization and wettability are obtained by direct plasma exposure, so the required treatment times for obtaining a rather narrow pattern on the surface of PS are well below half a minute. Still, the interface between the highly polar surface area and the non-affected area is rather sharp even after prolonged treatment. The thickness of the interface where the water contact angle drops from 95° to about 65° is roughly 1 mm. Even narrower interfaces can be obtained using VUV radiation only. In this case, the required treatment time is much longer, about a few minutes, but the central part of the spot assumes a very high concentration of polar functional groups. The polymer surface treated by radiation only is rich in functional groups of high oxygen content, such as carboxyl and carbonate. In contrast, the functionalities obtained by direct plasma treatment are significantly less pronounced. The differences in surface chemistry between direct plasma treatment and VUV radiation was explained by etching of the polymer surface upon interaction with different plasma species and thus simultaneous removal of the well-oxidized surface film.

## Figures and Tables

**Table 1:** XPS surface composition of PS covered with the MgF_2_ optical window and exposed to Ar atmospheric pressure plasma jets (APPJ) at various treatment times.

Treatment Time	C	N	O	O/C
0 s	97.9		2.1	0.02
30 s	85.8	1.0	13.2	0.15
60 s	80.2	1.4	18.4	0.23
180 s	70.6	2.0	27.4	0.39
600 s	67.4	1.7	30.8	0.46

**Table 2:** XPS surface composition of PS exposed directly to Ar APPJ at various treatment times.

Treatment Time	C	N	O	O/C
0 s	97.9		2.1	0.02
30 s	78.4	1.1	20.5	0.26
60 s	79.5	0.8	19.7	0.25
180 s	77.6	0.6	21.8	0.28
600 s	78.7	0.5	20.8	0.26

**Table 3:** XPS evolution of functional groups of PS treated by VUV radiation versus treatment times. For comparison, direct plasma treatment of PS for the longest treatment time is also shown.

Treatment Conditions	Treatment Time (s)	C–C	C–O	C=O	O=C–O	O–(CO)–O	π–π*
N/A	0	93					7
Covered with MgF_2_	30	91.0	5.3	0.8	1.2	0.4	1.3
Covered with MgF_2_	60	74.3	12.6	7.0	4.6	1.3	0.2
Covered with MgF_2_	180	62.5	16.6	9.0	7.4	4.2	0.1
Covered with MgF_2_	600	47.9	16.7	12.8	12.9	9.7	0.04
Direct APPJ treatment	600	78.4	11.0	3.4	1.8	3.6	1.9
